# Case Report: Integrative naturopathic approach for the management of sequestered lumbar disc herniation with neurological impairments: a case series with two year follow up

**DOI:** 10.3389/fpain.2024.1367683

**Published:** 2024-05-31

**Authors:** Sunil Paudel, Chethana A. M. Paudel, Karishma Silwal

**Affiliations:** ^1^ Department of Integrative Medicine, Spark International Health Resort, Kathmandu, Nepal; ^2^ Department of Naturopathy, Sant Hirdaram Medical College of Naturopathy and Yogic Sciences, Bhopal, India

**Keywords:** lumbar disc herniation, disc sequestration, conservative treatments, naturopathy, case series

## Abstract

Lumbar Disc Herniation (LDH) is a common condition, and contemporary pain research emphasizes the importance of adopting a comprehensive biopsychosocial perspective in pain treatment for positive clinical outcomes. Integrated Naturopathy and Yoga (INY) is a non-invasive medical system that takes a holistic and patient-centric approach to healing diseases. However, there is limited evidence on the effectiveness of INY, particularly in managing Sequestered LDH. We present two cases of patients experiencing radicular low back pain, lower limb weakness, and neuro-claudication who opted for conservative naturopathic management with INY. Following the INY treatments, both patients reported gradual relief from lower back pain, radicular pain, and neurological deficits. These findings are significant and contribute valuable evidence, suggesting that INY could be a viable therapeutic approach for managing sequestered LDH. This represents the first report on a non-invasive method for resolving sequestered LDH by utilizing INY.

## Introduction

Lumbar disc herniation (LDH) is a prevalent condition, impacting a range of 5–20 cases per 1,000 adults (1). LDH includes five recognized subtypes: bulging discs, focal protrusions, broad-based protrusions, extrusions, and sequestrations, with the latter being the most severe form (2). Sequestrations are the free fragments of nucleus pulposus and annulus fibrosus separated from the intervertebral disc in patients with LDH that may lead to severe symptoms, and neurological deficits, increasing the likelihood of surgical intervention (3). Existing evidence suggests a high natural tendency for sequestrations to regress, highlighting the promising effectiveness of conservative management (4, 5). A prospective cohort study showed that although operated patients initially had better pain and disability score improvements, there were no significant differences compared to patients with spontaneous regression by the 6th month (2).

Conservative management for disc herniation includes addressing ergonomics, postural care, counseling, home-based exercises, physical therapy, and the use of medicines (6). Additionally, spinal manipulation has been reported to be successful in inducing regression of LDH (7, 8), further broadening the spectrum of non-surgical options available. Nevertheless, these approaches, while valuable, may not fully address the complex biopsychosocial factors involved in pain management. Contemporary pain research underscores the crucial role of adopting a comprehensive perspective in treatment to achieve favorable clinical outcomes (9).

In response to these limitations, Integrated Naturopathy and Yoga (INY) emerge as a promising alternative. INY represents a non-surgical system of medicine that adopts a holistic and patient-centric approach to healing diseases (10). This approach incorporates a range of treatment modalities including counseling, manipulative therapy, hydrotherapy, therapeutic fasting, diet therapy, yoga therapy, heliotherapy, physiotherapy, acupressure, and acupuncture (11). Such a comprehensive method has not only demonstrated promising effects in alleviating pain and enhancing the functional quality of life in other musculoskeletal disorders but has also shown efficacy in comparison to physical therapy for managing non-specific low back pain (11–13). However, the available evidence on the effectiveness of INY in the management of sequestered LDH is currently limited. This prompted the presentation of two cases of sequestered LDH managed with INY in this study. The inclusion of INY in the management of sequestered LDH, therefore, offers a novel avenue for treatment that potentially mitigates the necessity for surgical interventions and aligns with the growing emphasis on holistic, patient-centered care.

The National Institute for Health and Care Excellence (NICE) guidelines (14), along with recommendations from the North American Spine Society (NASS) (15) and Spine surgery and related research guidelines (16), suggest surgical intervention only for the cases where conservative management proves ineffective or in situations of severe cauda equina syndrome and lower limb weakness. In line with both patients’ preference for a non-surgical approach and backed by evidence of successful outcomes for sequestered LDH through conservative management (4, 17); an INY treatment plan was designed.

## Case 1

**Figure 1:**
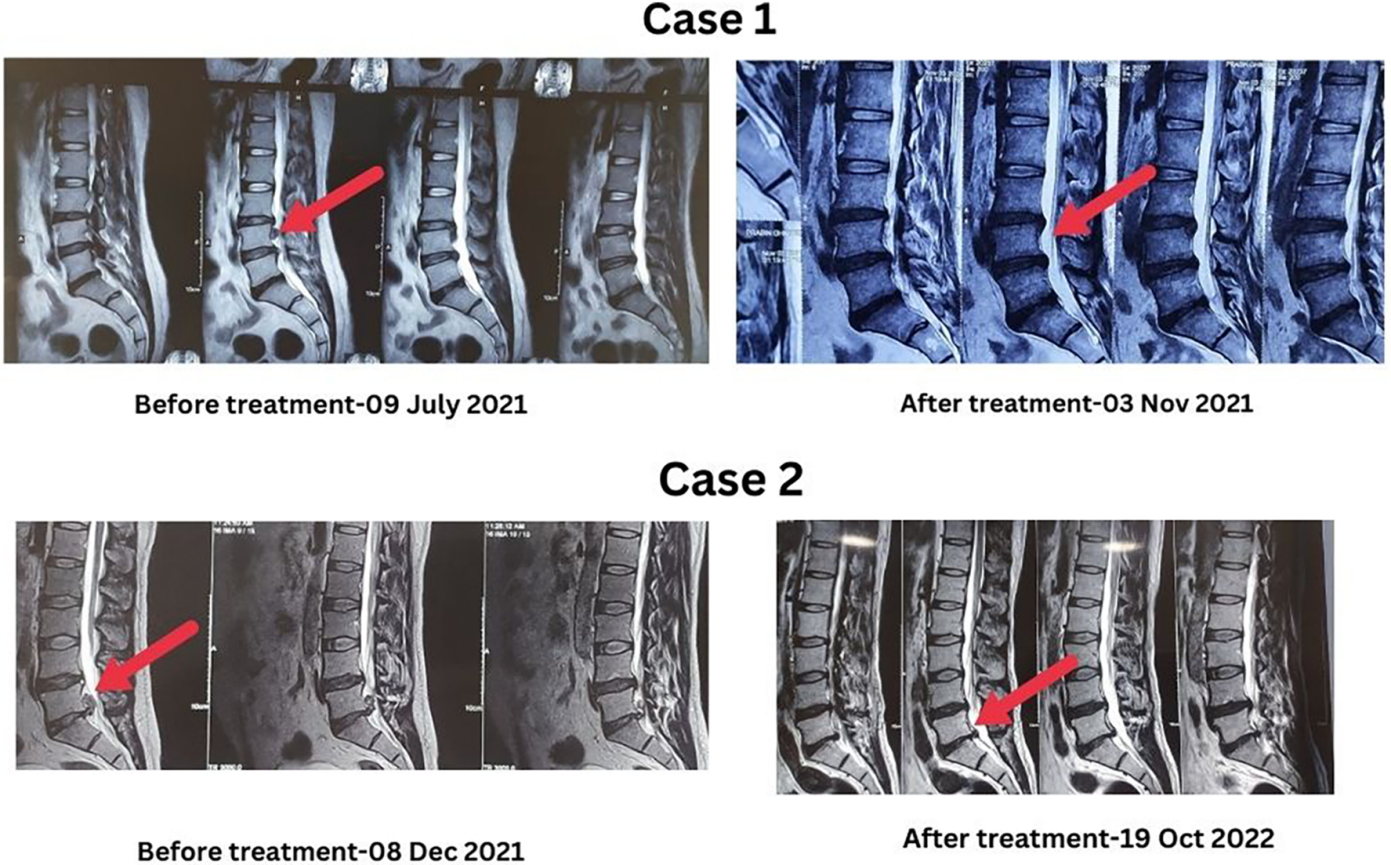
MRI changes before and after the treatments.

A 38-year-old man visited our clinic with complaints of radiating back pain, weakness in his right lower limb, and neuro-claudication. Physical examination revealed muscle weakness in his right extensor hallucis longus (grade 1/5) and dorsiflexors (grade 1/5). He faced difficulty walking, and there was a considerable impact on his activities of daily living (ADL). The sensation was diminished in the right L5 dermatome. Bowel and bladder functions were normal, the straight leg raising (SLR) test was positive at 60 degrees in the right leg, and reflexes were diminished (+1). MRI of the lumbar spine indicated herniated discs at L3/L4 and L4/L5, along with a sequestered LDH at L3/L4 with caudal migration. He was taking NSAIDs, adjuvants (pregabalin), multivitamins (neurase), vitamin D and calcium tablets. Opting against surgery due to his sister's history of failed back surgery syndrome, the patient chose conservative treatment at our integrative naturopathic clinic. He received treatment (see Supplementary File S1) on an outpatient basis. He started having some relief within a few days of treatments. Following four months of INY, the patient reported gradual and significant relief from radiating lower back pain and neurological deficits. Subsequent examinations, including physical examination and the straight-leg raising test, showed normal results with MRI showing resolution of the sequestered LDH (see Table 1 and Figure 1). Medications were gradually tapered, and the patient received advice on postural care, work ergonomics, and lifestyle changes during follow-up visits.

**Table 1:** Prognosis chart.

Cases	Parameters	Before treatment	After treatment	Follow up
Case 1		09 July 2021	03 Nov 2021	02 Dec 2023
	MRI scan	*L3-L4 Disc Extrusion, caudal migration and sequestration	*No sequestration	–
		*L4-5 minor LDH	L4-5 minor LDH persistent	
	NPRS	6	1	0–1
	Spinal flexibility	Diminished	Normal	Normal
	SLR test	Positive at 60 degrees in right leg	Negative	Negative
	Reflexes	Knee jerk: 1+	Knee jerk: 2+	Knee jerk: 2+
	Muscle power of affected muscle			
	Right Dorsiflexors	1/5	4-/5	5/5
	Right Extensor hallucis longus	1/5	4-/5	5/5
	Heel walking	Unable to walk	Better	Normal
	Toe walking	Normal	Normal	Normal
	Sensory	Diminished on right L5 dermatome	Normal	Normal
	Muscle tone	Normal	Normal	Normal
	Muscle bulk	Normal	Normal	Normal
	Bowel/bladder involvement	None	None	None
	Saddle anesthesia/paresthesia	None	None	None
	Functional ability	Unable to walk and work for more than 10 min	Able to walk and work with intermittent rest	Functionally active and normal
Case 2		08 Dec 2021	19 Oct 2022	02 Dec 2023
	MRI scan	**L5-S1 Disc sequestration with cranial migration	*No sequestration	–
		*L4-5 minor LDH	*L4-5 minor LDH persistent	
		*L5-S1 minor Retrolisthesis and disc protrusion	*L5-S1 minor Retrolisthesis and disc protrusion: slightly better	
	NPRS	7	2	0
	Spinal flexibility	Diminished movements	Normal	Normal
	SLR	Positive at 60 degrees on the right leg and 45 degrees on the left leg	Positive at 70 degrees on the right leg and 60 degrees on the left leg	Negative
	Reflexes	Knee/heel jerk 2+	Knee/heel jerk 2+	Knee/heel jerk 2+
	Muscle power of affected muscle			
	Left plantar flexors	3+/5	5/5	5/5
	Heel walking	Normal	Normal	Normal
	Toe walking	Unable	Better	Normal
	Sensory	Diminished on left S1 dermatome	Normal	Normal
	Muscle tone	Normal	Normal	Normal
	Muscle bulk	Normal	Normal	Normal
	Bowel/bladder involvement	None	None	None
	Saddle anesthesia/paresthesia	None	None	None
	Functional ability	On total bed-rest	Neuroclaudication after walking more than 10 min	Functionally Active and normal
			Able to walk and work with intermittent rest	

NPRS, numeric pain rating scale; SLR, straight leg raising; L3, lumbar 3; L4, lumbar 4; L5, lumbar 5; S1, sacral 1; LDH, lumbar disc herniation.

## Case 2

A 39-year-old man presented with a similar complaint of back pain radiating to the left lower limb, accompanied by claudication. Physical examination revealed muscle weakness in the left Plantar Flexors (grade 3/5). Like the previous case, he experienced difficulty walking, significantly impacting his ADL. The sensation was diminished on the left S1 dermatome. Bowel, and bladder functions were normal, but the SLR test was positive at 60 degrees in the right leg and 45 degrees in the left leg. MRI of the lumbar spine indicated herniated discs at L4/L5 and L5/S1, along with a sequestered LDH at L5/S1. This sequestered LDH caused cranial migration, resulting in spinal stenosis and compression of the cauda equina and transversing nerve roots. The patient was taking NSAIDs, adjuvants (pregabalin), vitamin B12, and B1 tablets. Despite doctors recommending surgery, he chose conservative management. After 15 days of inpatient treatments with complete bed rest followed by out-patient sessions and follow-ups; (see Supplementary File S1) the patient reported relief from radiating lower back pain and neurological deficits. Following 10 months of INY, the patient demonstrated substantial improvement in symptoms and the MRI showed minor persistence of LDH but showed resolution of the sequestered fragment (see Table 1 and Figure 1).

## Discussion

Both cases in the present study showed significant long-term improvement in symptoms of LDH as well as regression of sequestered LDH utilizing INY. This aligns with existing evidence that sequestered LDH can naturally regress without resorting to surgery as reported by Gugliotta et al. (6). A systematic review done by Chiu et al. found that spontaneous regression rates differed depending on the type of disc herniation; 96% for sequestration, 70% for extrusion, 41% for protrusion, and 13% for bulging. They concluded that sequestered and extruded LDH serves as a predictive factor for regression (5). Another randomized control trial by Weinstein et al. reported that patients in both the surgery and the non-surgical treatment groups improved substantially over 2 years emphasizing the preference for conservative management as the primary approach for sequestered LDH and associated complications (17, 18).

The likely mechanism behind the regression of sequestered discs involves an inflammatory response triggering immune cell-mediated degradation and neovascularization (19). Macrophages play a crucial role in enhancing phagocytosis of the herniated tissue and breaking it down using lysosomal enzymes (20). Kang et al. demonstrated that herniated discs release high levels of matrix metalloproteinases, nitric oxide, interleukins 6, and prostaglandin E2 (21). This indicates that the primary mechanism behind resolving LDH, especially the sequestered LDH are attenuating the inflammation, matrix remodeling, and shrinkage of nucleus pulposus back into the intervertebral space due to gradual dehydration and retraction (19, 22, 23). Oktay et al. (24) observed that a decrease in the herniation ratio is linked with clinical improvement, while Kong et al. (21) found that patients may experience symptom relief even if their disc herniation does not show radiological improvement. While both studies were retrospective and included patients who opted not to undergo surgery, this could introduce potential bias. However, more studies are required to evaluate and correlate spontaneous disc regression with clinical outcomes.

INY is a holistic healing system that operates based on the Healing Power of Nature (Vis Medicatrix Naturae) (25), and views inflammation as a process necessary to restore normal bodily function (26). INY approach advocates supporting the body's healing process by creating a conducive environment through elements such as rest, different therapeutic modalities, a proper diet, correct posture, and a positive mindset (25). An earlier study by Nair et al. has reported the positive impact of modalities such as hydrotherapy, massage, acupuncture, diet therapy, sun exposure, and yoga in reducing pain and improving the quality of life in patients with musculoskeletal disorders (11). The present study also used a similar approach in line with naturopathic principles that are aimed at nurturing the natural progression of inflammation and assisting holistic healing. There are systematic reviews and randomized control trials supporting the effectiveness of individual modalities such as acupuncture (27), yoga (28), psychotherapy (29), postural care and physical therapy (30) in managing non-specific low back pain. Nevertheless, there was still a gap in understanding the effectiveness of these modalities in integration, especially in the case of sequestered LDH. This case series serves as the foundation for further exploration.

We also observed a decrease in radiating low back pain, alongside improvements in sensations, muscle power, flexibility, and functional movements. Both cases had no return of symptoms throughout the two-year follow-up period, indicating the long-term efficacy of INY-based lifestyle treatments. The first patient's sequestered disc resorbed within 4 months, and the second patient within 8 months, with no reported adverse events. The period of resolution appeared to be shorter with INY compared to previous findings, where the average duration was 9 months (4), indicating that INY may have a role in accelerating LDH resorption.

While these findings are compelling and contribute valuable evidence for considering INY as a viable therapeutic approach in managing sequestered LDH, the study has limitations. First, as a case series with only two cases, generalization is limited. Second, the observed regression may be natural or a cumulative effect of treatment and natural regression. Therefore, future randomized control trials with adequate power and sample size are warranted to validate the efficacy of INY in sequestered LDH management. Nonetheless, this is the first report on a novel non-surgical approach to successfully treating sequestered LDH as well as neurological deficits associated with it.

## Patient’s perspective

Both patients conveyed satisfaction with their progress after opting for a non-surgical and cost-effective therapy to address their conditions.

## Data Availability

The original contributions presented in the study are included in the article/Supplementary Material, further inquiries can be directed to the corresponding author.

## References

[1] Dydyk AM Massa RN Mesfin FB. Disc Herniation. St. Petersburg, FL: StatPearls (2023). Available online at: https://www.ncbi.nlm.nih.gov/books/NBK441822/ (accessed November 24, 2023)

[2] Sucuoǧlu H Barut AY. Clinical and radiological follow-up results of patients with sequestered lumbar disc herniation: a prospective cohort study. Med Princ Pract. (2021) 30:244–52. 10.1159/000515308 33601393 PMC8280406

[3] Li ST Zhang T Shi XW Liu H Yang CW Zhen P Lumbar disc sequestration mimicking a tumor: report of four cases and a literature review. World J Clin Cases. (2022) 10:2883. 10.12998/WJCC.V10.I9.2883 35434096 PMC8968809

[4] Macki M Hernandez-Hermann M Bydon M Gokaslan A McGovern K Bydon A. Spontaneous regression of sequestrated lumbar disc herniations: literature review. Clin Neurol Neurosurg. (2014) 120:136–41. 10.1016/J.CLINEURO.2014.02.013 24630494

[5] Chiu CC Chuang TY Chang KH Wu CH Lin PW Hsu WY. The probability of spontaneous regression of lumbar herniated disc: a systematic review. Clin Rehabil. (2015) 29:184–95. 10.1177/0269215514540919 25009200

[6] Gugliotta M Da Costa BR Dabis E Theiler R Jüni P Reichenbach S Surgical versus conservative treatment for lumbar disc herniation: a prospective cohort study. BMJ Open. (2016) 6:e012938. 10.1136/BMJOPEN-2016-012938 28003290 PMC5223716

[7] Chu EC-P Sabourdy E. Non-surgical restoration of L3/L4 disc herniation. Cureus. (2023) 15:e40941. 10.7759/CUREUS.40941 PMC10368486 37496528

[8] Chu EC-P Yau KH-Y Bellin DL. An L2/3 disc herniation-related L5 radiculopathy. Curr Health Sci J. (2023) 49:129–33. 10.12865/CHSJ.49.01.129 37780195 PMC10541075

[9] Rhon DI Fritz JM Greenlee TA Dry KE Mayhew RJ Laugesen MC Move to health-a holistic approach to the management of chronic low back pain: an intervention and implementation protocol developed for a pragmatic clinical trial. J Transl Med. (2021) 19:1–13. 10.1186/S12967-021-03013-Y/FIGURES/3 34407840 PMC8371880

[10] Snider P Zeff J. Unifying principles of naturopathic medicine origins and definitions. Integr Med (Encinitas). (2019) 18:36. . 32549831 PMC7219457

[11] Nair PMK Silwal K Keswani J Kriplani S Khan V Maheshwari A Management of polyneuropathy using yoga and naturopathic medicine in India: recommendations for future research and clinical practice. Frontiers in Pain Research. (2023) 4:1264450. 10.3389/FPAIN.2023.1264450/BIBTEX 37954066 PMC10634222

[12] Szczurko O Cooley K Busse JW Seely D Bernhardt B Guyatt GH Naturopathic care for chronic low back pain: a randomized trial. PLoS One. (2007) 2:e919. 10.1371/JOURNAL.PONE.0000919 17878954 PMC1976391

[13] Paudel SK. Efficacy of yoga therapy in chronic low back pain-A critical review. Sense. (2012) 2:187–95. UDC: 233.852.5Y:611.88.959.

[14] UK, National Guideline Centre. Overview | Low back pain and sciatica in over 16s: assessment and management | Guidance | NICE (n.d.).

[15] Kreiner DS Hwang SW Easa JE Resnick DK Baisden JL Bess S An evidence-based clinical guideline for the diagnosis and treatment of lumbar disc herniation with radiculopathy. Spine J. (2014) 14:180–91. 10.1016/J.SPINEE.2013.08.003 24239490

[16] Sakai D. The essence of clinical practice guidelines for lumbar disc herniation, 2021: 4. Treatment. Spine Surg Relat Res. (2022) 6:329. 10.22603/SSRR.2022-0045 36051678 PMC9381073

[17] Weinstein JN Tosteson TD Lurie JD Tosteson ANA Hanscom B Skinner JS Surgical vs nonoperative treatment for lumbar disk herniation: the spine patient outcomes research trial (SPORT): a randomized trial. JAMA. (2006) 296:2441. 10.1001/JAMA.296.20.2441 17119140 PMC2553805

[18] Saal JA Saal JS. Nonoperative treatment of herniated lumbar intervertebral disc with radiculopathy. An outcome study. Spine (Phila Pa 1976). (1989) 14:431–7. 10.1097/00007632-198904000-00018 2718047

[19] Cunha C Silva AJ Pereira P Vaz R Gonçalves RM Barbosa MA. The inflammatory response in the regression of lumbar disc herniation. Arthritis Res Ther. (2018) 20:251. 10.1186/S13075-018-1743-4 PMC6235196 30400975

[20] Kobayashi S Meir A Kokubo Y Uchida K Takeno K Miyazaki T Ultrastructural analysis on lumbar disc herniation using surgical specimens: role of neovascularization and macrophages in hernias. Spine (Phila Pa 1976). (2009) 34:655–62. 10.1097/BRS.0B013E31819C9D5B 19333096

[21] Kang JD Stefanovic-Racic M McIntyre LA Georgescu HI Evans CH. Toward a biochemical understanding of human intervertebral disc degeneration and herniation. Contributions of nitric oxide, interleukins, prostaglandin E2, and matrix metalloproteinases. Spine (Phila Pa 1976). (1997) 22:1065–73. 10.1097/00007632-199705150-00003 9160463

[22] Teplick JG Haskin ME. Spontaneous regression of herniated nucleus pulposus. AJR Am J Roentgenol. (1985) 145:371–5. 10.2214/AJR.145.2.371 3875236

[23] Hu C Lin B Li Z Chen X Gao K. Spontaneous regression of a large sequestered lumbar disc herniation: a case report and literature review. J Int Med Res. (2021) 49:3000605211058987. 10.1177/03000605211058987 PMC8649452 34812080

[24] Oktay K Ozsoy KM Dere UA Cetinalp NE Arslan M Erman T Spontaneous regression of lumbar disc herniations: a retrospective analysis of 5 patients. Niger J Clin Pract. (2019) 22:1785–9. 10.4103/NJCP.NJCP_437_18 31793490

[25] Zeff J Snider P Myers S. Naturopathic model of healing—the process of healing revisited. Integr Med (Encinitas). (2019) 18:26–30. . 32549828 PMC7219459

[26] Wu YS Chen SN. Apoptotic cell: linkage of inflammation and wound healing. Front Pharmacol. (2014) 5:74705. 10.3389/fphar.2014.00001 PMC3896898 24478702

[27] Paley CA Johnson MI. Acupuncture for the relief of chronic pain: a synthesis of systematic reviews. Medicina 2020. (2019) 56:6. 10.3390/MEDICINA56010006 PMC7023333 31878346

[28] Holtzman S Beggs RT. Yoga for chronic low back pain: a meta-analysis of randomized controlled trials. Pain Res Manag. (2013) 18:267. 10.1155/2013/105919 23894731 PMC3805350

[29] Yang J Lo WLA Zheng F Cheng X Yu Q Wang C. Evaluation of cognitive behavioral therapy on improving pain, fear avoidance, and self-efficacy in patients with chronic low back pain: a systematic review and meta-analysis. Pain Res Manag. (2022) 2022:4276175. 10.1155/2022/4276175 PMC8957446 35345623

[30] García-Moreno JM Calvo-Muñoz I Gómez-Conesa A López-López JA. Effectiveness of physiotherapy interventions for back care and the prevention of non-specific low back pain in children and adolescents: a systematic review and meta-analysis. BMC Musculoskelet Disord. (2022) 23:1–14. 10.1186/S12891-022-05270-4/TABLES/4 35366847 PMC8976404

