# Supplementary material for: Case Report: Integrative naturopathic approach for the management of sequestered lumbar disc herniation with neurological impairments: a case series with two year follow up
Source: Front Pain Res (Lausanne). 2024 May 31;5:1367683. doi: 10.3389/fpain.2024.1367683 (PMC11176481; doi:10.3389/fpain.2024.1367683)
Supplement: Supplementary file 1 [file Table1.docx]

**Supplementary file 1: Integrative Naturopathy Treatment Plan**

| **Interventions** | **Description** | **Duration and Dosage**  **Case 1** | **Duration and Dosage**  **Case 2** | **Rationale** |
| --- | --- | --- | --- | --- |
| Counseling | Patients had opted against surgery. He was well informed of consequences. He was also made aware of prospective evidence-based alternatives based on earlier studies. Counseling was done to explore an integrative naturopathic conservative management, care and home treatments. | First day: 45 mins to 1 hour.  Subsequent Weeks: A counseling session (15 to 20 mins) every week for a month.  Then one every month (5 to 15 mins). | First day: 45 mins to 1 hour.  Subsequent Weeks: A counseling session (15 to 20 mins) every week for a month.  Then one every month (5 to 15 mins). | To inform about the natural spontaneous resorption of sequestered LDH  To provide education on alternative approaches that can be considered.  To educate about the innate healing mechanisms rooted in naturopathic principles. |
| Postural Care | Education on strict postural maintenance and self-help in day to day life. | To be observed all the time | To be observed all the time. | To reduce the cumulative load on the lower back/disc. |
| Dietary changes | A well-balanced meal plan comprising nuts, seeds, eggs, tofu, fermented foods, millets, vegetables, fruits, salads, and sprouts. They were advised to refrain from consuming junk foods, fast foods, sugars, and processed foods. | Around 2000 kcal in a day | Around 2000 kcal in a day | To alleviate inflammation and provide essential nutrients for the healing process. |
| Naturopathy treatments | Full body /Partial massages  Steam bath | 30 mins daily  Once weekly  Treatments were administered for one month on an outpatient basis, followed by daily massage at home for 3 months. | 30 mins daily  Once weekly  Treatments were provided for 15 days on an in-patient basis, followed by alternate-day sessions for a month on out-patient basis, with subsequent visits limited to full body massages and occasional steam bath only. | To support the natural pain management and enhance the spontaneous natural regression of sequestered LDH.  To boost endorphin release and keep the whole body fit and healthy. |
| Electrotherapy | TENS  IFT  UST    Heating Modalities  Electrical muscle stimulation (EMS) | 20 mins  15 to 20 mins  5 to 10 mins (1 MHZ)  15 to 20 mins once a day  EMS using Galvanic Current by placing pads on the dorsiflexor and using a pen electrode on the extensor hallucis longus.  Treatments were administered for one month on an outpatient basis then occasionally. | 20 mins  15 to 20 mins  5 to 10 mins (1 MHZ)  15 to 20 mins X 2 times a day  EMS using Galvanic Current by placing pads on the plantar-flexors.  Treatments were provided as inpatient sessions for 15 days, followed by alternate-day sessions for a month, and then weekly or fortnightly sessions for the next three months on an outpatient basis. | To alleviate pain, reduce muscle stiffness, and enhance flexibility and strength in joints, nerves, and muscles.  To reduce radiation and parasthesia.  To improve muscle power. |
| Yoga and Exercises | Pawanmuktasan Series  Uttanapadasan Half Crunches Abdomen sets | Initially performed 10 repetitions per day, and was gradually increased to 50 repetitions per day.  (Up to 2 times a day) | Initially performed 5 repetitions per day, and was gradually increased to 30 repetitions per day.  (Up to 3 times a day) | To enhance flexibility and strength in joints and muscles.  To assist natural regression of sequestered LDH.  Promote local circulation.  Reduce stress  To maintain/improve muscle power |
|  | Ankle Pumps Heel Walking Toe Walking | Initially performed 20 repetitions per day, and was gradually increased to 100 repetitions per day.  (Up to 2 times a day) | Initially performed 5 repetitions per day, and was gradually increased to 50 repetitions per day.  ( Up to 2 times a day) |  |
| Acupuncture | Local analgesic points were used | 30 minutes daily for 10 days  Then on & off | 30 minutes daily for 10 days  Then on and off | Bring out positive biochemical and hormonal homeostasis necessary for muscle, bones, and joint health  To promote circulation locally, release muscular tightness and stiffness  To enhance natural pain management. |
| Home based treatments and lifestyle changes | Sun Bath  Home-massage  Education on posture, sleep hygiene  Exercises, yoga and pranayama | 20 mins daily  15-20 mins  -  10-30 mins daily  Or  Whenever Possible | 20 mins daily  15-20 mins  -  30-40 mins daily  Or  Whenever Possible | To emphasize sustained health improvement  To enhance vitamin D levels for bone, muscle, and nerve health naturally  To manage stress  To maintain unloading to the disc or low back  To maintain spinal health to prevent subsequent LDH. |

**TENS**-Transcutaneous Electrical Nerve Stimulation, **IFT-** Interferential Therapy, **UST**-Ultrasound Therapy, **MHZ**-Megahertz, **Mins**-Minutes, **kcal**- Kilocalories, **LDH**-Lumbar disc herniation.
